# Charges associated with pediatric head injuries: a five year retrospective review of 41 pediatric hospitals in the US

**DOI:** 10.5249/jivr.v5i1.205

**Published:** 2013-01

**Authors:** Brian D. Robertson, Charles E. McConnel, Sally Green

**Affiliations:** ^*a*^Children’s Medical Center Dallas, 1935 Medical District Dr., Dallas, Texas 75235.; ^*b*^UT Southwestern Medical Center, 5323 Harry Hines Blvd., Dallas, Texas 75390.

**Keywords:** Brain injury, Causes, Cost, Children

## Abstract

**Background:**

Brain injuries are a significant public health problem, particularly among the pediatric population. Brain injuries account for a significant portion of pediatric injury deaths, and are the highest contributor to morbidity and mortality in the pediatric and young adult populations. Several studies focus on particular mechanisms of brain injury and the cost of treating brain injuries, but few studies exist in the literature examining the highest contributing mechanisms to pediatric brain injury and the billed charges associated with them.

**Methods:**

Data were extracted from the Pediatric Health Information System (PHIS) from member hospitals on all patients admitted with diagnosed head injuries and comparisons were made between ICU and non-ICU admissions. Collected data included demographic information, injury information, total billed charges, and patient outcome.

**Results:**

Motor vehicle collisions, falls, and assaults/abuse are the three highest contributors to brain injury in terms of total numbers and total billed charges. These three mechanisms of injury account for almost $1 billion in total charges across the five-year period, and account for almost half of the total charges in this dataset over that time period.

**Conclusions:**

Research focusing on brain injury should be tailored to the areas of the most pressing need and the highest contributing factors. While this study is focused on a select number of pediatric hospitals located throughout the country, it identifies significant contributors to head injuries, and the costs associated with treating them.

## Introduction

Unintentional injuries are the fourth leading cause of death for all age groups,^1,2^ and the leading cause of death for individuals under the age of 19.^1^ Moreover, head and brain injuries account for approximately one-third of all injury deaths across all age groups,^4,5^ and are the most common cause of morbidity and mortality in children,^6-8^ representing almost ninety percent of all pediatric injury deaths.^9^

Brain injuries vary in severity and are generally measured on the Glasgow Coma Scale (GCS), a 12-point scale ranging from 3-15 measuring a person’s degree of lucidity and responsiveness. The GCS is the sum measurement of scores assigned to a person’s papillary reaction to light, motor movement response, and verbal appropriateness.^9^ Scores falling between 12 and 15 are generally considered mild brain injuries, scores between 9 and 12 are generally considered moderate brain injuries, and scores of 8 and below are generally considered severe brain injuries.^6,10^ No distinction of severity was made within the scope of this study beyond admission to the Intensive Care Unit of their respective hospitals, representing or serving as a proxy for more severe brain injuries.

The cost of treating traumatic brain injuries including hospital, extended care, other medical care, disability, and lost income was estimated at approximately $38 billion in 1985.^11^ More recent studies focusing specifically on pediatric brain injury estimated $1 billion annually in pediatric hospital charges,^3^ and almost $1 billion in billed charges in the year 2000.^12^ The biggest contributors to brain injuries in the pediatric population are falls and motor vehicle crashes.^13-16^

Previous literature on pediatric brain injury has focused on a wide variety of topics ranging from hospitalization trends and billing^3,11,12^ to the clinical management of head injuries,^8^ but very little data has been published from a public heath standpoint on the general health problem of head injuries in the pediatric population. Some studies have been published on the brain injuries across all age groups,^11,17^ but few studies specifically focus on the highest contributing mechanisms of head injury and the cost of treating head injuries based on the mechanism of injury.^13^ The purpose of this study was to ascertain and present a general public health perspective on head and brain injuries in the pediatric population, and to determine some degree of the economic burden of pediatric brain injuries. 

## Methods

Following Institutional Review Board approval, data were accessed via the Children’s Health Corporation of America (CHCA; Shawnee Mission, KS) Pediatric Health Information System (PHIS) database, a unique database which contains resource utilization data from 42 freestanding children’s hospitals and a capture rate of eighteen percent. Participating hospitals are located in non-competing markets of 27 states plus the District of Columbia. CHCA and participating hospitals jointly assure data quality and reliability as described previously.^18,19^The PHIS database includes demographic information such as patient age, gender, payor status, resident town size, and location of treating hospital. Injury information includes date of admission, discharge date and cause of injury and the outcome information includes length of stay, discharge disposition, and total billed charges.

Patients were included in this study if they were diagnosed with a head injury using ICD-9 codes 800, 801, 803, 804, 850-854, and 959.01 between 1/1/2006 and 12/31/2010, and were flagged if they were treated in an Intensive Care Unit. The selected ICD-9 codes include fractures of the skull vault, fracture of the skull base, other and unqualified skull fractures, multiple fractures involving the skull or face with other bones, concussions, cerebral lacerations and contusions, subarachnoid, subdural, and extradural hemorrhage following injury, other intracranial hemorrhage following injury, intracranial injury of other nature, and unspecified injuries to the head or neck. The first ICD-9 code listed for the patient was used to determine whether the head injury was a primary or secondary injury.

**E-codes and Mechanism of Injury**

External cause of injury codes (E-codes) were used to determine the specific cause of injury for each of these patients. Patients coded with medical accidents, medication reactions, and poisonings were excluded from this analysis. 

E-code groupings basically followed the recommendations of the MMWR report,^20^ but were coded to see causal factors at a finer level. Research on E-codes show difficulty in using broad-ranging categories^21^ due to coding complications.^22^ For this reason, we broke the E-codes into more narrowly-focused categories that allowed for better comparisons between groups. In classifying E-codes, the first E-code listed in the diagnosis list was the E-code that was used to identify the cause of injury. The breakdown for all E-codes used in this study is found in Appendix A.

**Injury Type: Accidental and Non-Accidental Injuries**

Because non-accidental injuries generally have more severe injuries, longer hospital stays, and worse outcomes compared to accidental injuries,^23^ all violence-related non-accidental injuries were broken into a separate category called “abuse/assault,” and will be examined in more detail separately. Using E-codes, all injuries were broken down into accidental and non-accidental injuries regardless of the mechanism of injury. Those with an unknown causal factor were listed as unknown injuries. Self-inflicted injuries were kept as a separate category and not included in the non-accidental grouping.

**Town Size and Geographical Area**

For geographical mapping purposes, the United States was categorized into five different geographical regions. To help maintain confidentiality of the hospitals, each region has at least two CHCA member hospitals. Town sizes were defined using the Second Edition Rural and Urban Commuting Area Code (RUCA 2) data, which bases town sizes from zip codes.^24^ Towns were then broken up using previously published data on town sizes^4,23^ to identify urban towns, large towns, small towns, and isolated towns. Due to confidentiality issues, the individual zip codes (which are encrypted in PHIS) were not provided to the researchers, but were linked to the RUCA 2 data by CHCA staff.

**Billed Charges**

The billed charges captured in this dataset are the actual charges billed by the hospital for the entire hospital stay. All billed charge data were converted to 2010 dollar values using a standard conversion calculation. The total billed charges for 2010 were divided by 1, 2009 values were divided by 0.967, and divided by 0.9370, 0.90376, and 0.8655 for 2008, 2007, and 2006 values, respectively.^25^ With a capture rate of eighteen percent, national projections were approximated by multiplying the total billed charges by five.

All data was analyzed using SPSS version 18 (SPSS, Chicago, IL).

## Results

**Demographics and Geographical Distribution**

In all, 39,657 patients were included in the study, with 273 being excluded for medical accidents, medication errors, and poisonings. The remaining 39,384 were included in the analysis. Demographic information and regional breakdowns are found in Table 1 , and injury information is found in Table 2.

**Table:** Table 1: **Patient Demographic Information**

	ICU Admits	Non-ICU Admits	Total
Total N	11,245	28,139	39384*
Male	7,210 (64%)	17,977 (63%)	25,187 (64%)
Female	4,024 (35%)	10,150 (36%)	14,174 (36%)
Mean Age (in years)	6.32 (± 5.6)	6.26 (± 5.7)	6.28 (± 5.7)
Regional Distribution			
Deep South	2,540 (23%)	6,960 (25%)	9,500 (24%)
Midwest	2,972 (26%)	6,307 (22%)	9,279 (24%)
Northeast	1,880 (17%)	5,561 (20%)	7,441 (19%)
Southwest	2,274 (20%)	4,755 (17%)	7,029 (18%)
West	1,579 (14%)	4,556 (16%)	6,135 (15%)
Town Size			
Urban	8,494 (75%)	22,571 (80%)	31,065 (79%)
Large Town	1,250 (11%)	2,446 (9%)	3,696 (9%)
Small Town	671 (6%)	1,244 (4%)	1,915 (5%)
Isolated Town	409 (4%)	814 (3%)	1,223 (3%)
Not Listed	421 (4%)	1,064 (4%)	1,485 (4%)
Payor Source			
Government Insurance	4,901 (44%)	11,317 (40%)	16,218 (41%)
Private Insurance	3,064 (27%)	8,931 (32%)	11,995 (30%)
Other Insurance	1,846 (16%)	4,660 (17%)	6,506 (17%)
Not Listed	1,434 (13%)	3,231 (11%)	4,665 (12%)

Data collected from 39,384 cases nationwide Data represent n (%) or mean (± standard deviation) *Sex was not listed in 23 patients

**Table:** Table 2: **Injury and Accident Information**

	ICU Admits	Non-ICU Admits	N
Injury			
Primary	8,336 (74%)	21,725 (77%)	30,061 (76%)
Secondary	2,909 (26%)	6,414 (23%)	9,323 (24%)
Injury Type			
Accidental	7,145 (64%)	20,461 (73%)	27,606 (70%)
Non-Accidental	1,306 (12%)	2,072 (7%)	3,378 (9%)
Unknown	146 (1%)	346 (1%)	492 (1%)
Not Listed	2,648 (23%)	5,260 (19%)	7,908 (20%)
Discharge Information			
Home	9,261 (82%)	26,870 (95%)	36,131 (92%)
Other	680 (6%)	521 (2%)	1,201 (3%)
Died	767 (7%)	406 (1%)	1,173 (3%)
Skilled Facility	326 (3%)	162 (1%)	488 (1%)
Home Health Service	211 (2%)	180 (1%)	391 (1%)

Data collected from 39,384 cases nationwide

All patients included in this study were under the age of 18, with an average age of 6.28 years (standard deviation of 5.7). Over the five year period, sixty-four percent of the population was male (n = 25,187). The majority of the patients in the sample had government health insurance (n = 16,218), thirty-one percent were on private insurance (n = 11,995), almost seventeen percent were on “other insurance” (n = 6,506), and almost twelve percent did not have listed insurance status (n = 4,665).

**Figure 1: United States Regional Map:**
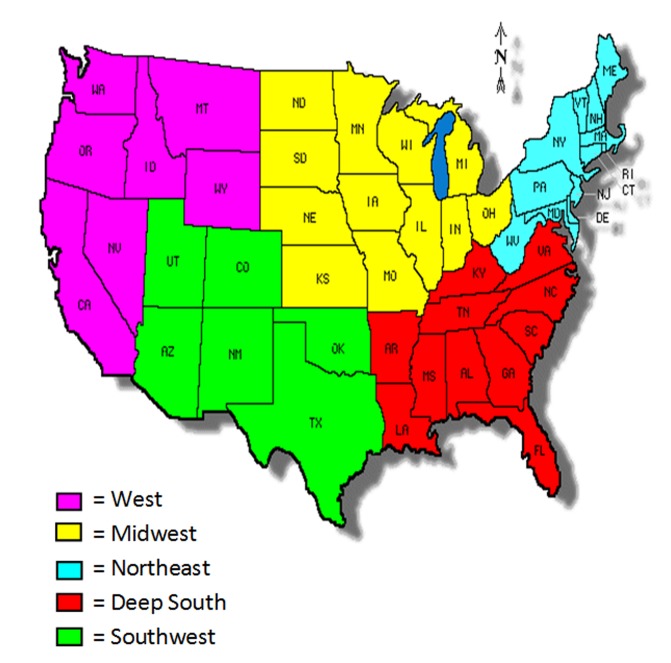


The majority of the patients lived in urban towns (79%, n = 31,065), followed by large towns (9%, n = 3,696), small towns (5%, n = 1,915), and then isolated towns (3%, n = 1,223). Resident town size was not listed in almost four percent of the sample (n = 1,485). A map of the regional breakdown is found in Figure 1.

**Injury Status, Outcome, and Accident Information**

Over three-quarters of the patients captured in this dataset were diagnosed with a head injury as the primary diagnosis (n = 30,061), and almost one-quarter of the patients had diagnosed head injuries secondary to another injury (n = 9,323). 

Almost seventy percent of the patient population was injured through an accidental mechanism (n = 27,606), and almost nine percent were injured through non-accidental means (n = 3,378). Approximately twenty-one percent of the patients had an unknown causal type or the cause was not listed (n = 492 and 7,908, respectively).

A majority of the patients were discharged home (92%, n = 36,131), with “other” as the next most common discharge disposition (3.0%, n = 1,201). Three percent of the population died (n = 1,173), and one percent went to a skilled facility at discharge (n = 488). One percent of the patient population were also discharged into a Home Heath Service (n = 391).

Chi-square analysis further yields significant differences for type of diagnosed head injury (primary or secondary), type of injury, and discharge disposition (p < 0.01). Patient Injury Status and Outcome information can be found in Table 2.

**Mechanism of Injury and Billed Charges**

Twenty percent of the population did not have an identifiable e-code listed (n = 7,908). However, the most common mechanisms of injuries were falls (n = 11,535), all motor vehicle-related crashes (n = 8,251), and abuse/assaults (n = 3,299). These three mechanism areas represent almost fifty-nine percent of the total injuries. In this instance, motor vehicle-related crashes included motor vehicle collisions, motor vehicle accidents, motor pedestrian collisions, and “other motor vehicle accidents.”

Overall, the total billed charge for these patients was $1.73 billion dollars over the past five-year period. Approximately six percent of the population had no billed charges listed (n = 2,354), and one patient had a billed credit. Excluding these patients from the analysis, the average billed charges in treating these patients was $46,784 (Md $18,369). The average length of ICU stay for these patients was 5.3 days (Md 2 days), ranging from 1 to 1,534 days. The most expensive billed charge was $7.8 million.

Overall, government insurance was billed an average of $52,357 per patient (Md $20,104), private insurance was billed an average of $43,763 per patient (Md $17,615), other insurance was billed an average of $40,929 per patient (Md $16,244), and those with no insurance listed were billed an average of $42,998 per patient (Md $17,510).

For the top ten most common injuries, the total billed charges and the average charges for those injuries are found in Table 3, Table 4 and Table 5, respectively. Motor vehicle collisions and crashes, falls, and assaults/abuses not only account for over half of the injuries, but also account for half of the billed charges over the five-year period. Motor vehicle crashes account for $406.3 million in billed charges over the five-year period, with falls accounting for $244.3 million, and assault/abuse accounting for another $219.5 million in billed charges. Combined, these three areas account for almost $1 billion in billed charges over the five-year period. The top ten most expensive injuries by billed charges are found in Table 4, and the top ten most expensive injuries by average cost per injury are found in Table 5.

**Table:** Table 3: **Top 10 Most Common Head Injury Causes between 2006 and 2010**

Rank	Cause	ICU Admits	Non-ICU Admits	Total
1	Fall	2,229 (20%)	9,303 (33%)	11,535 (29%)
2	MVA	2,305 (21%)	4,205 (15%)	6,510 (16%)
3	Assault/Abuse	1,277 (11%)	2,022 (7%)	3,299 (8%)
4	Struck by or Against an Object	503 (4%)	1,465 (5%)	1,968 (5%)
5	Unspecified Accident	424 (4%)	1,393 (5%)	1,817 (5%)
6	MPC	632 (6%)	1,109 (4%)	1,741 (4%)
7	Pedal Cycle Accident	345 (3%)	1,128 (4%)	1,473 (4%)
8	Sports	234 (2%)	873 (3%)	1,107 (3%)
9	ATV/Snowmobile Accident	272 (2%)	696 (2%)	968 (2%)
10	Animal Injury	139 (1%)	337 (1%)	476 (1%)

Data collected from 39,384 cases nationwide

**Table:** Table 4: **Top 10 Most Expensive Head Injury Causes Between 2006 and 2010 (gross)**

Rank	Cause	Total Injuries	Total Billed Charges	National Projection*
1	MVA	6,510	$377,735,908	$1,888,679,540
2	Fall	11,535	$229,080,110	$1,145,400,550
3	Assault/Abuse	3,299	$206,039,197	$1,030,195,985
4	MPC	1,741	$111,031,461	$555,157,305
5	Unspecified Accident	1,817	$63,574,970	$317,874,850
6	Struck By or Against Object	1,968	$59,405,074	$297,025,370
7	ATV/Snowmobile	968	$36,749,239	$183,746,195
8	Pedal Cycle Accident	1,473	$29,710,149	$148,550,745
9	Sports Injury	1,107	$19,589,211	$97,946,055
10	Animal Injury	476	$18,165,366	$90,826,830

Data collected from 39,384 cases nationwide *PHIS capture rate is 18%, and National Projection was calculated by multiplying Total Billed Charges by 5.

**Table:** Table 5: **Top 10 Most Expensive Head Injury Causes Between 2006 and 2010 (average)**

Rank	Cause	Total Injuries	Average Billed Charge	National Projection*
1	Aircraft Accident	6	$191,437	$957,185
2	Other Asphyxiation	7	$181,685	$908,425
3	Firearms/Explosives	135	$100,062	$500,310
4	Overexertion	11	$81,106	$405,530
5	Stab/Slice/Piercing	54	$79,333	$396,665
6	MPC	1741	$67,373	$336,865
7	Assault/Abuse	3299	$65,119	$325,595
8	Neglect	26	$62,545	$312,725
9	MVA	6510	$61,711	$308,555
10	Watercraft Accident	27	$56,303	$281,515

Data collected from 39,384 cases nationwide

It should be reiterated that almost twenty percent (n = 7,549) of the study sample had no E-codes listed. These patients account for $461.4 million of the total sum, and an average billed charge of $61,124 per patient. These totals rank first, and tenth in the total billed charges and average billed charge, respectively. 

## Discussion

Head and brain injuries are a significant contributor to morbidity and mortality in the pediatric and young adult populations. Moreover, they are complex injuries that can be sustained through a number of different mechanisms and causal factors. While many articles discuss the incidence rates of brain injuries as a whole, very little has been published looking at the different causal factors and the costs associated with them.^13^

This results of this study are similar to another study that identifies motor vehicle collisions and falls as the highest contributors to head injury in the pediatric population.^13^ Previous research documented the top five causes of head injuries as motor vehicle crashes, falls, assaults, other transports accidents, and being struck by or against an object.^13^ However, motor vehicle crashes were lower in this study (16.4% vs. 38.9%), and falls were higher (29.1% vs. 21.2%).^13^ The difference in motor vehicle crashes may be due to the age constraints in this study, and inability for most pediatric hospital patients to drive.

This study shows a marked difference in the total cost of healthcare from previous studies. Where Bowman et al (2008) estimated $1 billion annually in hospital costs for injured children, this study reports annual charges approximately one-third lower. The biggest contributing factor to this difference is the selective use of only 41 pediatric hospitals instead of data incorporating nationwide totals. While missing charges can also account for smaller charge numbers, billing information was documented in ninety-four percent of the patients included in this dataset. However, a more significant factor is the use of billed charges in this study and not the overall healthcare costs to these patients. While the overall costs represent a better global picture of the economic burden of treating these injuries, this study is able to show more direct-care costs to insurance companies, taxpayers, and hospitals.

This study reports approximately $1.7 billion in billed charges over a five-year period, again markedly lower than another study focusing on hospital utilization in children with traumatic brain injury that reports almost $1 billion in total charges for the year 2000 alone.^12^

However, the database utilized in this study has a capture rate of eighteen percent versus previous studies utilizing nationwide totals. With an eighteen percent capture rate, we can project a national total for billed charges at $9.4 billion over a five-year period. This then converts to $1.8 billion in annual estimated billed charges nationwide in 2010 dollars. While higher than previous studies, this may be due to specialized trauma centers and higher level of care that may be associated with treating head injuries at these centers. Where Schneier et al (2006) reports an average billed charge of $20,325 (Md $8,056), our study reports average billed charges more than twice as high for the total sample. However, non-ICU admissions had an average billed charge of $24,444 (Md $13,224) in this study.

The payor source is also different from previous research. Schier et al (2006) reports sixty percent of the sample size having private insurance and only twenty-five percent covered under Medicaid. The number of patients with government insurance in our study is almost twice as high (41%) and the number of patients with private insurance is exactly half (30%).

Additionally, this study attempted to identify the mechanism of injury related to traumatic brain injury. However, this dataset may have captured both brain and facial injuries, not the injuries related specific to the brain. Clearer and more specific inclusion criteria focusing on intracranial injuries would be more beneficial in the future, but this is one of the limitations of conducting studies using large databases. This study also made no distinction between the severity of the brain injury, and captured all patients listed in the database fitting the inclusion criteria. Data in this study identified patients who were admitted to an ICU, which implies more severe injuries. However, without GCS scores we cannot accurately distinguish or quantify the severity of the injuries in this dataset. 

We were also unable to determine whether injuries were localized only to the head, or included multi-system/multi-organ injuries. Multi-organ and mult-system injuries would result in longer hospital stays, higher billed charges, and worse outcomes.

E-code data has the potential to be a great tool for global causal factors, but the vast number of diagnoses a patient may have in a single encounter coupled with missing E-codes make research in this area very difficult. This study alone identified almost forty-thousand patients with ICD-9 codes designating head injuries, but almost twenty percent of the sample did not have an identifiable E-code listed. Missing E-codes can have a significant impact on the outcome of any research conducted using these codes. It is further suggested that any research utilizing E-codes should be more narrowly-focused so that the factors involved in the E-code are analyzed, not the E-code itself.

Another problem with conducting retrospective research on databases is relying on coders to correctly code data. Previous research on the accuracy of E-codes in falls found that forty-six percent of the charts listing an E-code for unspecified falls could be assigned a more specific E-code based on the information documented in the patients’ charts.^26^ As ICD-9 codes are diagnostic codes used for primarily for billing purposes instead of the clinical management of patients, the validity of database information is questionable, and its application is limited. Additionally, assigning accurate E-codes may be difficult, particularly in determining accidental and non-accidental injuries, based on medical records. Without the ability to review specific charts, we are unable to verify the accuracy of the E-code data.

Another limitation of this study is the inability to capture zip codes of where injuries actually occur. This type of data can generally only be extracted through prospective studies, hospital-specific studies, or other database studies where this information is documented. Residential information may be useful, but research focusing on injuries should focus on the location of the injury whenever possible.

This dataset may also lend itself to selection bias. While many pediatric institutions focus on and treat childhood injuries, not all children under the age of 18 will be seen at pediatric medical centers as older children may be treated at adult hospitals. Although this dataset included and captured patients under the age of 18, those who were treated at adult facilities would not be captured in this dataset. This then points to an overall younger population, smaller catchment, and lower estimate of the projected billed charges for the pediatric population.

Despite the limitations, this study lends a significant contribution to the public health literature, namely in identifying an expanded listing of the top mechanisms of injury to the largest cause of morbidity and mortality in the pediatric population. Instead of focusing on and exploring a specific mechanism, we are able to examine the causes of brain injury as a whole, and the billed charges associated with them. While the billed charges do not represent a complete picture of the cost burden for treating these injuries, it captures some semblance of the magnitude of hospital costs based on the mechanism of injury, which contributes to understanding the breadth and scope of these injuries. Being able to understand brain and head injuries, particularly in terms of the specific mechanism of injury and the greatest contributing factors leading to the causes of head injuries, better prepares public health practitioners and injury prevention professionals to identify the most significant contributors to brain and head injuries. Only with this understanding will we be truly effective at reducing the incidence or prevalence of head injuries in children.

**Table:** Appendix A.**E-code Table**

Mechanism	E-codes
Motor Vehicle Accidents (MVC)	E810-813, E815-819, E822-825, E829, E846-848, E929.0, E988.5
Motor Pedestrian Collisions (MPC)	E814
ATV/Snowmobile	E820-821
Watercraft Accident	E830-831, E838
Aircraft Accident	E840-841, E844
Railway Accident	E800-801, E805, E807
Assault/Abuse	E928.3, E960, E963-969
Self-Inflicted	E950, E953, E955, E957-959
Neglect	E904
Fall	E804, E834-835, E843, E880.0, E880.1, E880.9, E881.0, E882.0, 883.2, E883.9, E884, E885, E886.0, E886.9, E888, E929.3, E987
Drowning/Submersion	E832, E910
Other Asphyxiation*	E911-913, E983
Animal Injury	E827-828, E905-906
Burn/Scald	E837, E890-891, E893-894, E924, E926, E988.2
Crush Injuries	E918
Environmental Accidents	E900-901, E907-909, E928.8
Explosion/Electrocution	E921, E925
Firearms/ Explosives	E922-923, E985, E997
Home Injuries	E013, E849.7
Late Effects of Injuries	E929.1, E989
Machinery Accident	E919-920.1
Pedal Cycle Accident	E826
Overexertion	E927
Sports Accident	E006-007, E917.0, E917.5
Winter Sports	E003
Stab/Slice/Pierce	E914-915, E920.3, E920.4, E920.8, E920.9
Struck by or Against Object	E916, E917.1, E917.2, E917.3, E917.4, E917.6, E917.7, E917.8, E917.9
Other Accidents	E029.2, E883.0, E973
Unspecified Accidents	E000, E029.9-030, E849.0, E849.3, E849.4, E849.5, E849.6, E849.8, E849.9, E887, E928.9, E929.9, E983.8, E987.0, E988.8-988.9

*Includes choking on food, choking on non-food items, other mechanical asphyxiation, and strangulation not able to be determined as accidental.
